# Autophagy Proteins in Viral Exocytosis and Anti-Viral Immune Responses

**DOI:** 10.3390/v9100288

**Published:** 2017-10-04

**Authors:** Christian Münz

**Affiliations:** Viral Immunobiology, Institute of Experimental Immunology, University of Zürich, Winterthurerstrasse 190, CH-8057 Zürich, Switzerland; christian.muenz@uzh.ch; Tel.: +41-44-635-3716

**Keywords:** Epstein Barr virus, Varizella Zoster virus, poliovirus, coxsackie B virus, MHC class I molecules, MHC class II molecules, LAP (LC3 associated phagocytosis)

## Abstract

Autophagy-related (Atg) gene-encoded proteins were originally described for their crucial role in macroautophagy, a catabolic pathway for cytoplasmic constituent degradation in lysosomes. Recently it has become clear that modules of this machinery can also be used to influence endo- and exocytosis. This mini review discusses how these alternative Atg functions support virus replication and viral antigen presentation on major histocompatibility (MHC) class I and II molecules. A better understanding of the modular use of the macroautophagy machinery might enable us to manipulate these alternative functions of Atg proteins during anti-viral therapies and to attenuate virus-induced immune pathologies.

## 1. Introduction

Autophagy is a cellular degradation process that delivers cytoplasmic constituents to lysosomes for nutrient recycling [1]. It consists of at least three pathways, which either allow for substrate acquisition directly at the lysosomal or late endosomal membrane, as in the case of micro- and chaperone-mediated autophagy, or engulf parts of the cytoplasm in a double-membrane surrounded vesicle, named autophagosome, which then fuses with lysosomes. The latter pathway is called macroautophagy and last year’s Nobel laureate Yoshinori Ohsumi identified the first 15 genes that contribute to the molecular machinery of this pathway [2]. These autophagy-related (atg) genes, of which now more than 30 have been identified, fall into four functional groups [1]. The Unc-51 Like Autophagy Activating Kinase 1 (ULK1)/Atg13 complex integrates the nutritional status of the cell via inhibitory phosphorylation by the mammalian target of rapamycin (mTOR). It itself regulates the activity of a lipid phosphorylating complex, namely the vacuolar vrotein vorting (VPS) 34/Beclin-1 phosphatidylinositol (PI) 3 kinase complex, by phosphorylation. The resulting PI3P marks membranes that then recruit the LC3 lipidation complex, which couples in a ubiquitin-like fashion Atg8 orthologues (LC3A, LC3B1, LC3B2, LC3C, GABA receptor-associated protein (GABARAP), GABARP-L1 and GABARAP-L2) to phosphatidylethanolamine in membranes that are elongated to become the outer and inner autophagosome membrane. The Atg8 orthologues most likely contribute to this elongation and recruit substrates into autophagosomes. Finally, the closed autophagosome fuses with lysosomes involving Plekstrin homology domain-containing protein (PLEKHM) 1, syntaxin 17 and the fusion machinery that is also in part used by late endosomes [3,4]. In the resulting autolysosomes, the inner autophagosomal membrane and its cargo are degraded and nutrients recycled by transport into the cytosol.

These modules of the macroautophagy machinery (ULK1/Atg13 complex, VPS34/Beclin-1 complex, LC3 lipidation complex and autophagosome fusion complex) are also used for other cell biological processes that require the modulation of intracellular membranes [5]. In this review, I will mainly discuss regulation of endo- and exocytosis by Atgs. Phagosomes that endocytose toll-like receptor (TLR) agonists, immune complexes via Fc receptor binding and apoptotic cell debris via scavenger receptors like T-cell immunoglobulin mucin protein (TIM) 4 [6,7,8,9,10] recruit LC3 to the cytosolic side of their membrane. This requires the VPS34/Beclin-1 complex and the LC3 lipidation, but not the ULK1/Atg13 complex. In contrast to macroautophagy this LC3-associated phagocytosis (LAP) requires, however, the nicotinamide adenine dinucleotide phosphate (NADPH) oxidase (NOX2)-dependent production of reactive oxygen species (ROS) at the phagosomal membrane [7,10,11]. Depending on the cell type, LAP leads to enhanced degradation, substrate maintenance or fusion with specialized endosomal compartments [8,9,10]. Atg proteins are in addition involved in non-conventional secretion. Several substrates that lack signal peptides for co-translational insertion into the ER benefit from Atgs for their secretion. These include acyl-CoA-binding protein (Acb1) and interleukin 1β (IL-1β) [12,13,14,15,16]. In contrast to LAP, the molecular requirements for this pathway are just beginning to be elucidated, but it seems that the ULK1/Atg13, the VPS34/Beclin-1 and the LC3 lipidation complexes are involved in Atg-supported exocytosis [12,13,14]. In addition Golgi-associated proteins (GRASPs), like GRASP55, and the SNAP receptors (SNAREs) Sec22b, syntaxin 3 and 5, soluble NSF attachment protein (SNAP) 23 and SNAP29 seem to be required for secretion [13,14,16]. However, the exocytosed cargo might be contained within the autophagosome, in the space between the inner and outer autophagosomal membrane, or attached to the outer autophagosomal membrane [15,17,18]. Thus, Atg-supported exocytosis might be either a redirection of autophagosomes for fusion with the cell membrane rather than with lysosomes or an alternative use of LC3-conjugated membranes without autophagosome generation. In the following review, I will discuss how macroautophagy, LAP and Atg-supported exocytosis influence viral replication and viral antigen presentation on major histocompatibility complex (MHC) molecules.

## 2. Autophagic Envelope Acquisition by Herpes Viruses

**Figure 1:**
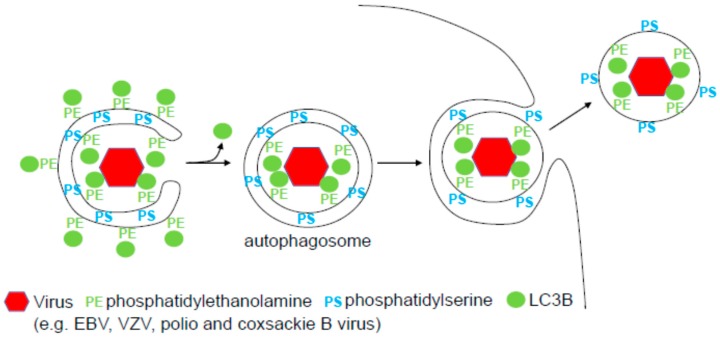
Viral exocytosis with autophagic membranes. Viruses, like some herpes and some picornaviruses, seem to sample LC3B coated membranes for their envelope, which are enriched in phosphatidylserine for scavenger receptor uptake.

Such redirected fusion with the plasma membrane and release of the inner autophagosomal membrane might be used by some herpes viruses [19] (Figure 1). Herpes viruses are enveloped large double-stranded DNA viruses that undergo two envelope acquisitions during lytic replication, allowing non-cytolytic release from infected cells [20]. The first envelope is generated by the budding of the herpes viral capsid, which is assembled in the nucleus of infected cells, through the inner nuclear membrane. This envelope is lost after fusion with the outer nuclear membrane and the capsid then acquires tegument proteins in the cytosol. Herpes viruses acquire their second envelope from ER and Golgi membranes and then travel surrounded by two membranes to the cell surface, where the outer membrane fuses with the cell membrane and the inner membrane becomes the final herpes virus envelope. Within the herpes virus family there are several members that seem to inhibit macroautophagy during lytic replication, often targeting Beclin-1 with their Bcl-2 homologues or herpes simplex virus (HSV) encoded infected cell protein (ICP) 34.5 [21,22]. These include the α-herpes virus HSV and the γ-herpes virus Kaposi sarcoma-associated herpes virus (KSHV). Interestingly, within the same subfamilies of herpes viruses (α and γ), two other viruses, varicella zoster virus (VZV) and Epstein Barr virus (EBV), utilize LC3-coated membranes for their second envelopes [23,24]. EBV was found to accumulate LC3-conjugated membranes upon induction of lytic replication in both B and epithelial cells [23]. Inhibition of the LC3 lipidation complex reduced the production of infectious viral particles, while rapamycin inhibition of mTOR, and the consequent activating of the ULK1/Atg13 complex, stimulates virus production [23,25]. Upon inhibition of LC3 lipidation, viral genomes accumulate in the cytosol, presumably lacking efficient second envelope acquisition [23]. Consistent with autophagic membranes contributing to the viral envelope, purified EBV particles contain lipidated LC3. Similarly, LC3-coupled membranes accumulate during VZV infection [26]. Inhibition of the VPS34/Beclin-1 complex with 3-methyladenine or the LC3 lipidation complex by Atg5 silencing compromises infectious virus production and viral glycoprotein (VZV gE) accumulation in cytoplasmic membranes. Furthermore, lipidated LC3 can also be found in purified VZV particles, and VZV gE partially colocalizes with LC3 in the cytoplasm [24]. These experiments suggest that viral glycoproteins get stabilized in LC3 coated membranes, which are then incorporated into the envelopes of EBV and VZV. If these enveloped capsids, however, then use the machinery for Atg-supported exocytosis that I have described above, this needs further investigation. Nevertheless, it is quite likely that the respective herpes viruses hijack such existing pathways of exocytosis that utilize parts of the macroautophagy machinery.

## 3. Atg Assisted Exocytosis of Picornaviruses

While herpes viruses select membranes from Golgi and ER, which are considered primary sites of autophagosome generation [27,28], and then travel in phenotypically autophagosome-like structures to the cell membrane [29,30], it is far less obvious how non-enveloped RNA viruses, such as the picornaviruses poliovirus, coxsackie B virus and rhinoviruses, benefit from autophagic membranes. Nevertheless, the first study that actually observed double-membrane-surrounded vesicle accumulation upon viral infection was performed with poliovirus [31]. Indeed, these accumulating membranes need the LC3 lipidation complex for their formation [32]. While some flaviviruses, like hepatitis C virus and chikungunya virus, use enlarged membrane compartments, which in part depend on the autophagic machinery for their RNA replication [33,34,35] and Norovirus RNA replication complexes are restricted by the LC3 conjugation complex [36], picornaviruses seem to use autophagic membranes for non-cytolytic spreading of their infection [37]. Indeed, poliovirus release was reported to require autophagosome maturation with acidification [38] and be inhibited by microtubule transport [39]. However, such viral exocytosis from double-membrane-surrounded cytoplasmic vesicles would result in viral particles surrounded by the inner autophagosomal membrane and picornaviruses are considered non-enveloped. In paradigm-shifting studies, it was, however, discovered that picornaviruses can exit cells in a non-cytolytic fashion as packages of multiple viral particles, surrounded by LC3-conjugated membranes [40,41] (Figure 1). Indeed, mature coxsackie B viruses were found in extracellular microvesicles containing lipidated LC3 [40]. This non-cytolytic release of poliovirus was dependent on the VPS34/Beclin-1 and LC3 lipidation complexes [41]. It resulted in extracellular microvesicles with around 20 viral particles surrounded by single LC3-coated membranes [41]. The benefit for non-enveloped picornaviruses to spread in extracellular microvesicles could be the non-cytolytic release, thereby maintaining virus production without host cell death for prolonged periods of time, and protection from host immunity, such as anti-viral antibodies, in the extracellular space. However, why is the sampling of autophagic membranes for these virus packages particularly attractive? One reason for this could be the particular lipid composition of autophagic membranes. In poliovirus-infected cells it was found that the membranes of the released viral particle-containing microvesicles are enriched in phosphatidylserine (PS) [41] (Figure 1). PS is also exposed on apoptotic bodies after programmed cell death and recognized by scavenger receptors on phagocytes for clearance of this cellular debris [42]. Indeed, blocking of two of these receptors, TIM and tyrosine-protein kinase receptor UFO (AXL), or of PS itself blocks efficient infection with picornaviruses [41,43]. Thus, picornaviruses like polio-, coxsackie B- and rhinoviruses, recruit LC3-coated PS-rich membranes for their non-cytolytic release as packages of viral particles in order to facilitate infection of scavenger receptor-expressing phagocytes. After uptake, these PS-containing membranes will be degraded followed by infection via the virus-specific receptors. However, as with herpes viruses, not all picornaviruses seem to use autophagic membranes in this fashion, because it was recently reported that Atg-independent budding into multivesicular bodies allows hepatitis A virus to acquire membranes for non-cytolytic release [44]. Therefore, as with herpes viruses, the future characterization of the molecular interactions of picornavirus proteins with the macroautophagy machinery might shed light on why some viruses exit cells with autophagic membranes and others do not.

## 4. Exo- and Endocytosis with Atg Modules for MHC Class I Antigen Presentation

**Figure 2:**
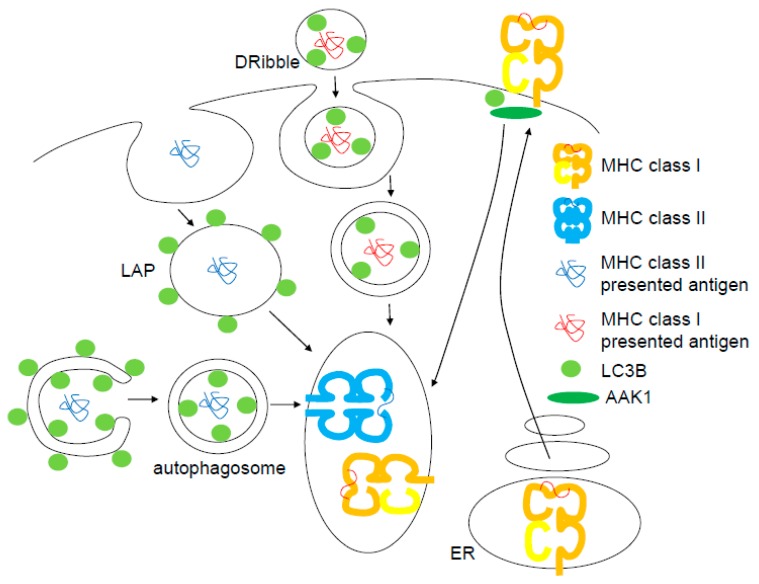
Atg8 orthologues regulate antigen presentation on MHC class I and II molecules. LC3B coated autophagosomes and phagosomes deliver intracellular and extracellular antigens for MHC class II presentation, respectively. LC3B positive DRibbles get processed for antigen presentation on MHC class I molecules and MHC class I molecules get internalized in an LC3B and adaptor associated kinase 1 (AAK1) dependent fashion.

A similar exocytosis of autophagic membranes might give rise to exosomes and indeed LC3 has been found in exosome preparations [45]. These vesicles might be superior antigen formulations for cross-presentation on MHC class I molecules to cytotoxic CD8^+^ T cells [46]. Indeed, compromising the VPS34/Beclin-1 or the LC3 lipidation complex inhibits the antigen transfer from tumor- and virus-infected cells to dendritic cells for efficient MHC class I presentation [47,48]. The generation and loading of these antigen-containing extracellular microvesicles can be enhanced by lysosome and proteasome inhibition, and the resulting vesicles are called defective ribosomal products containing autophagosome-rich blebs (DRibbles) [49]. Lysosome inhibition seems to promote their secretion and proteasome inhibition allows for their more efficient loading with defective ribosomal products, which are considered to be superior antigens for MHC class I loading (Figure 2). Such DRibble preparations have been successfully used for vaccination [49]. Thus, Atg-supported exocytosis allows also for antigen transfer to dendritic cells and their cross-presentation of such antigens on MHC class I molecules to cytotoxic CD8^+^ T cells.

This beneficial role of the macroautophagy machinery for cross-presentation is counterbalanced by its role in restricting MHC class I and MHC class I-like antigen presentation on the cell surface of antigen-presenting cells (APCs) by endocytosis [50,51]. Indeed, the net outcome of deficiency in Atg components of the LC3 lipidation complex in myeloid or dendritic cells is an elevated, often CD8^+^ T cell-mediated adaptive immune control or immunopathology [52,53]. Furthermore, deficiency of the LC3 lipidation complex in intestinal epithelium improves CD8^+^ T cell mediated immune surveillance of colorectal cancer development [54]. In part, this enhanced CD8^+^ T cell response to professional and other MHC class I APCs is due to the fact that antigen-presenting MHC class I molecules are stabilized on the cell surface of LC3 lipidation deficient cells [50]. This accumulation is due to decreased internalization that results from a poor recruitment of the adaptor-associated kinase 1 (AAK1) to MHC class I molecules in the absence of LC3 lipidation, and AAK1 facilitates MHC class I endocytosis for degradation (Figure 2). This results in increased priming of influenza A virus-specific CD8^+^ T cells and decreased infection associated viral titers and pathology in the absence of LC3 lipidation in dendritic cells. Similar to this regulation of classical MHC class I molecule endocytosis, the LC3 lipidation machinery also supports internalization of the non-classical MHC class I-like molecule, CD1d [51]. This leads to enhanced glycolipid presentation on CD1d to NKT cells and improved NKT cell-mediated immune control of the *Sphingomonas paucimobilis* infection. Therefore, lipidated LC3 might recruit parts of the internalization machinery for classical MHC class I and CD1d to these molecules on the cell membrane, which facilitates their internalization, but restricts CD8^+^ T and NKT cell responses, respectively.

## 5. Autophagy and LAP for MHC Class II Antigen Presentation

In a mechanism that might be related to the Atg-supported internalization of MHC class I molecules, the VPS34/Beclin-1 and LC3 lipidation complexes also regulate phagocytosis. This pathway is called LAP [8]. LAP was found to support MHC class II-restricted presentation of phagocytosed antigen to CD4^+^ helper T cells [10,55,56] and to restrict inflammation at the intestinal mucosa [57,58]. TLR2-engaging *Candida albicans* extract was found to be maintained for prolonged periods of time in human macrophages and dendritic cells before being presented to specific CD4^+^ T cell clones [10]. LC3B was found to be recruited to these *Candida*-containing phagosomes in a NOX2 function-dependent fashion. This LC3B coat was maintained on phagosomes for hours and deconjugated before fusion with lysosomes. In contrast, the C-type lectin receptor Dectin-1 phagocytoses also cargo by LAP in mouse macrophages [55]. This results in rapid lysosomal fusion and presentation on MHC class II molecules, presumably due to more efficient transport to lysosomes along microtubules [59] and/or more efficient fusion [3]. These studies suggest that the macroautophagy machinery without the ULK1/Atg13 complex can regulate endocytosis, improving antigen presentation of extracellular antigens on MHC class II molecules to CD4^+^ T cells (Figure 2).

In addition to this extracellular antigen processing via Atgs, intracellular antigens can also be presented via macroautophagy on MHC class II molecules [60] (Figure 2). Among these are also the macroautophagy proteins LC3B, GABARAP and GABARAP-L2 [61,62]. This intracellular self-antigen presentation seems to contribute to positive and negative CD4^+^ T cell selection in the thymus [63,64,65]. Furthermore, some viral proteins are presented on MHC class II molecules after macroautophagy [66,67,68], and mycobacterial pathogens have developed strategies to inhibit this pathway [69]. However, most impressively, intracellular antigens that are coupled to the N-terminus to LC3B get more efficiently presented on MHC class II molecules [64,70,71,72,73,74]. This enhanced presentation extends to influenza, melanoma, SIV/HIV and self-antigens, and can be achieved in dendritic, B, epithelial and tumor cells. Depending on the cell type and its capacity to present intracellular antigens on MHC class II molecules, CD4^+^ T cell recognition can be boosted up to 20-fold both in vitro and in vivo [70,74]. Thus, macroautophagy can transport intracellular antigens to MHC class II loading compartments for antigen processing and presentation to CD4^+^ T cells. This indicates that both extracellular and intracellular antigens benefit from the Atg core machinery of the VPS34/Beclin-1 and LC3 lipidation complexes for presentation on MHC class II molecules.

## 6. Conclusions and Outlook

The above-described studies reveal a modular composition of the macroautophagy machinery, in which just some of these modules can be used for endocytosis and exocytosis, in addition to cytoplasmic constituent degradation in lysosomes, which is the classical function of macroautophagy. It will be important to unravel how these modules are differentially recruited for these different tasks and how the resulting pathways might be individually therapeutically regulated. Viruses can show us the way to understand the underlying biological processes better, because they have most likely not developed separate pathways in order to use Atgs for their non-cytolytic release from infected cells, but hijack existing mechanisms of non-conventional secretion. Furthermore, it will be interesting to explore the possibility that LAP or Atg-supported endocytosis in general is also utilized by viruses during their entry.
